# The role of the Sunfrail tool in the screening of frailty and in integrated community-hospital care pathways: a retrospective observational study

**DOI:** 10.1007/s40520-021-01931-x

**Published:** 2021-07-24

**Authors:** Yari Longobucco, Fulvio Lauretani, Luciano Gionti, Sara Tagliaferri, Robbert Gobbens, Tomasz Kostka, Ernesto Palummeri, Mirca Barbolini, Marcello Maggio

**Affiliations:** 1 Department of Medicine and Surgery, University of Parma, Via Antonio Gramsci 14, 43126 Parma, PR Italy; 2 Geriatric Clinic Unit, Medicine-Geriatric-Rehabilitation Department, University-Hospital of Parma, Parma, Italy; 3 San Giuseppe Moscati Hospital, Caserta Local Health Trust, Caserta, Italy; 4 Faculty of Health, Sports and Social Work, Inholland University of Applied Sciences, Amsterdam, The Netherlands; 5 Zonnehuisgroep Amstelland, Amstelveen, The Netherlands; 6 Department Family Medicine and Population Health, Faculty of Medicine and Health Sciences, University of Antwerp, Antwerp, Belgium; 7 Department of Geriatrics, Medical University of Lodz, Healthy Ageing Research Centre (HARC), Lodz, Poland; 8 Galliera General Hospital, Genoa, Italy; 9 European Commission (DG Santè), Brussels, Belgium; 10 Catholic University of the Sacred Heart, Rome, Italy

**Keywords:** Ageing, Frailty, Primary care, Integrated care, Community nursing, Sunfrail

## Abstract

**Background:**

One of the most problematic expression of ageing is frailty, and an approach based on its early identification is mandatory. The Sunfrail-tool (ST), a 9-item questionnaire, is a promising instrument for screening frailty.

**Aims:**

- To assess the diagnostic accuracy and the construct validity between the ST and a Comprehensive Geriatric Assessment (CGA), composed by six tests representative of the bio-psycho-social model of frailty;
- To verify the discriminating power of five key-questions of the ST;
- To investigate the role of the ST in a clinical-pathway of falls’ prevention.

**Methods:**

In this retrospective study, we enrolled 235 patients from the Frailty-Multimorbidity Lab of the University-Hospital of Parma. The STs’ answers were obtained from the patient’s clinical information. A patient was considered frail if at least one of the CGAs’ tests resulted positive.

**Results:**

The ST was associated with the CGA’s judgement with an Area Under the Curve of 0.691 (CI 95%: 0.591–0.791). Each CGA’s test was associated with the ST total score. The five key-question showed a potential discriminating power in the CGA’s tests of the corresponding domains. The fall-related question of the ST was significantly associated with the Short Physical Performance Battery total score (OR: 0.839, CI 95%: 0.766–0.918), a proxy of the risk of falling.

**Discussion:**

The results suggest that the ST can capture the complexity of frailty. The ST showed a good discriminating power, and it can guide a second-level assessment to key frailty domains and/or clinical pathways.

**Conclusions:**

The ST is a valid and easy-to-use instrument for the screening of frailty.

## Introduction

The population progressive ageing trend and the increased demand for health and social care, coupled with countries reduced resources for health and social services, hampers the accessibility to healthcare for older persons [1]. One of the most problematic expression of populations’ ageing is the condition of frailty, a state of extreme vulnerability to sudden changes in health status induced by minimal stress events, which can cause an increase in adverse events [2, 3]. The prevalence of frailty in the elderly population presents a wide variability due to the numerous existing definitions [4]: in fact, there is no universally shared definition of frailty [3, 5]. The bio-psycho-social model considers it as a dynamic state that affects individuals with losses in multiple functional domains, increasing the risk of adverse events [5, 6]. This model is the most appropriate for identifying patients who could benefit from integrated care [7, 8] and to structure both health and social interventions aimed at counteracting the progression of frailty. The literature highlights how frailty is not an irreversible state, especially in the early stages [9]. There is now current agreement that integrated interventions are needed to slow down progression towards disability. Evidences of the positive effects of these interventions are growing [10–12]; in particular, since frailty is considered a multifactorial syndrome, the literature recommends that its screening should be followed by the Comprehensive Geriatric Assessment (CGA) to identify the causes and the appropriate interventions. This type of assessment has a multi-domain and a multidisciplinary nature, and it possibly has to include in addition to a Geriatrician, a specific-trained Nurse and a Social Worker. A systematic review demonstrated significant improvements in the management and outcomes of frail elderly when care models based on a CGA are applied [9].

There are several obstacles to the implementation of early interventions. First of all, there is no single definition of frailty, which is not well distinguished from disability. This issue has led to the existence of numerous tools for screening and detecting frailty, used at different stages of the treatment path [6, 13, 14]. In addition to the wide heterogeneity of the application contexts, a systematic review highlights the absence of a gold standard, and a series of limitations including the mono-dimensional approach, the extreme complexity, the self-administration and the detection of aspects related to disability [6].

It is evident that frailty requires a different approach, based on its early identification, centred on the person rather than on the disease and coordinated at the level of primary care through the integration of the various services. The CGA represents the best tool to respond to these characteristics, but it is unthinkable to integrate it into the routine practice of the primary care setting due to administrative and time constraints [15].

For these reasons, it is necessary to use a simple and agile tool, which considers frailty according to its multi-domain nature, allowing the identification of individuals who deserve a second level CGA. The Sunfrail tool (ST) has previously been shown as a valid instrument for assessing frailty [16] and has an excellent negative predictive value (NPV) of 84.6%, allowing to improve the appropriateness in the request of a CGA by the General Practitioner [17]. This tool, which was designed by an international multidisciplinary group and inspired on bio-psycho-social model, is composed by nine items related to the three domains of frailty (physical, cognitive, and social). One strength of the tool is that it can be used by multiple disciplines, primarily General Practitioners and Community Nurses, but also Social Workers, Caregivers, local Community Actors, and others. Its items generate alerts that lead to the activation of further assessments and/or specific care pathways [18].

The ST would allow us to improve the response to frailty, which is currently reactive to its late acute events, to a proactive and integrated care approach, that first identifies the possible needs and risk factors, and then intervenes by planning with the patient the most appropriate care pathway.

The primary aim of this study was to perform a diagnostic accuracy study between the ST and a second-level CGA’s judgement.

The secondary aims were:

- to assess the construct validity between the ST questions and the CGA’s tests;
- to verify the discriminating power of five questions of the ST, chosen because they investigate the same aspects assessed by some CGA tests;
- as history of falls is a predictor of future falls [19], we aimed to investigate the possible role of the ST in a clinical pathway of falls’ prevention.

## Materials and methods

### Population and setting

In this retrospective study, we enrolled 417 patients, visited at the Frailty-Multimorbidity Lab of the Geriatric Clinic Unit of University-Hospital of Parma. We excluded all the institutionalized and/or disabled patient, and we included only the first visits, resulting in a sample of 235 patients. The answers to the ST were retrospectively obtained from the patient’s clinical information, and collected with an interview conducted by a specialized nurse before the actual visit as a routine procedure. This operation was carried-out blindly to the judgement of frailty given by the CGA.

### Sunfrail tool description

The ST was developed following the methodology indicated in the literature for the creation of questionnaires [20–22], as part of a project funded by the third Health Program 2014–2020 of the European Commission.

The ST is composed by nine questions, as described in Table 1.

**Table 1:** Items of the Sunfrail tool

1	Do you regularly take 5 or more medications per day?	Physical domain
2	Have you recently lost weight such that your clothes have become looser?	Physical domain
3	Your physical state made you walking less during the last year?	Physical domain
4	Were you visited by your family doctor during the last year?	Physical domain
5	Have you fallen one or more times over the last year?	Physical domain
6	Have you experienced memory decline during the last year?	Cognitive domain
7	Do you feel lonely most of the time?	Social domain
8	If necessary, can you count on someone close to you?	Social domain
9	Have you had any financial difficulties in facing dental care and health care costs during the last year?	Social domain

Questions 1 through 5 concern the physical domain, question number 6 belongs to the cognitive domain, and the last three questions refer to the social domain of frailty.

To find the optimal cut-off value, the total score of the ST was calculated. A point was assigned for each affirmative answer, except for questions 4 and 8, where the point was assigned in case of a negative answer.

To better investigate the individual domains of frailty captured by the ST, the single-domains’ score were calculated, adding up the points of the item 1–5 for the physical domain, 7–9 for the social domain and with the affirmative answer to the item n°6 representing the positivity of frailty in the cognitive domain.

### Comprehensive geriatric assessment

Since there is no tool to be used as a gold standard and considering its high predictive ability of adverse health outcomes, it was decided to use the CGA as a reference measure [6]. The team, composed by a Geriatrician and a specialized Nurse, has collected information on age, sex, body mass index (BMI), and used tools of different extraction, described below, considered as gold standard in literature [23], to guarantee a multi-dimensional evaluation. All the questionnaires were interviewer-administered. According to the bio-psycho-social model of frailty, which states that “…frailty is a dynamic state affecting an individual who experiences losses in one or more domains of human functioning (physical, psychological, social)…” [24], a patient was considered frail if at least one of these tests or questionnaire resulted positive.

### Mini mental state examination

The Mini Mental State Examination (MMSE) evaluates the cognitive state through 30 questions with an overall score ranging from 0 to 30 [25], with a cut-off of ≤ 24 points as an indication of the presence of possible cognitive impairment [26].

### Short physical performance battery

The Short Physical Performance Battery (SPPB) represents one of the main physical tests able to predict mobility disability, and it represents a proxy of frailty [27]. Briefly, it is composed by three tasks: the ability to maintain balance in tandem, semi-tandem, and side-to-side positions, the walking speed at usual pace over 4 m and the ability to standing from a seated position for 5 times. The score ranges from 0 to 12, and a participant was considered frail with a score between 3 and 9 (included) [28].

### Handgrip test

Handgrip strength is a non-invasive marker of muscle strength, and low grip strength is associated with poor healthcare outcomes [29]; we referred to the European Working Group on Sarcopenia in Older People (EWGSOP2) revised cut-offs [30], which considers a cut-off for low strength of < 27 kg in men and < 16 kg in women. This test was performed with a Jamar dynamometer in a seated position, with three attempts for each hand.

### Five item Geriatric Depression Scale

The 5-item Geriatric Depression Scale (GDS-5) is used as a screening tool for identifying depression in older adults; a score ≥ 2 suggests the presence of a depressive state [31].

### Mini nutritional assessment-short form

The Mini Nutritional Assessment-Short Form (MNA-SF) is a short, validated nutritional screening tool composed by a subset of six questions from the full MNA. It is able to identify older individuals as well-nourished (MNA-SF score between 12 and 14), at risk of malnutrition (MNA-SF score between 8 and 11) or malnourished (MNA-SF score between 0 and 7) [32]. In this study, we considered the test positive with a score ≤ 11.

### Eating assessment tool-10 items

The Eating Assessment Tool-10 items (EAT-10) is a screening tool used for the evaluation of dysphagia risk. It’s composed by 10 questions, and the score ranges from 0 to 40; a cut-off ≥ 3 suggests the presence of swallowing difficulties [32].

### Statistical analysis

Data were analyzed with Stata 13 (Statacorp LLC, College Station, Texas, USA).

To verify the appropriateness of the sample size, a post hoc power analysis was conducted. Assuming a type-1 error of 0.05 and a type-2 error of 0.20, the minimum sample size resulted in 171 individuals. A descriptive analysis was conducted to describe the characteristics of our sample; continuous variables were reported as mean and standard deviations (SD) when normally distributed, alternatively as median and interquartile range. Dichotomous variables were reported as frequency and percentages. Normality was assessed with the Shapiro–Wilk test; since almost all the variables showed a non-parametric distribution, differences in means were evaluated with the Mann–Whitney *U* test, except for the Sunfrail total score and the Sunfrail physical domain score, which were assessed with the Student *t* test. The diagnostic accuracy was investigated using the receiver operating characteristic (ROC) curve analyses, with the judgement of frailty by the CGA as outcome, to estimate the area under the curve (AUC), sensitivity, and specificity and for each cut-point of ST’s score. After identifying the potential cut-off values, the positive predictive value (PPV) and the negative predictive value (NPV) were calculated. To analyse the construct validity of ST, we examined the correlations between the ST total and its three domains with the CGA’s tests, expressed in Spearman correlation coefficients, because of the non-parametric distribution of the CGA’s variables. As reported by a previous study [16], it was expected that the ST domains would show highest correlations with their corresponding domains of the CGA, and lowest correlations with the other domains. For the same reasons as above, we used the Mann–Whitney *U* test to verify any difference in key CGA tests based on the responses to five questions of the ST; the questions were the number 2, 3, 5, 6, and 7. Finally, we used logistic regression models to deepen the relationship between the question n°5 and the tests of the physical domain of CGA, SPPB, and Handgrip Test, adjusted by sex, age, BMI, and MMSE.

## Results

### Characteristics of the participants

The 46.4% of sample was composed by women (*N* = 109), and presented a mean age of 81.7 ± 7.0 years and a mean BMI of 27.3 ± 4.9 kg/m^2^. On the basis of the CGA, 210 (89.4%) patients were classified as frail: the judgement of frailty was given if at least one of the tests used was positive. The average MMSE score was 22.7 ± 4.7, suggesting a trend of the sample to a condition of mild cognitive impairment. Regarding the physical function, 128 patients (54.5%) presented a Grip Strength impairment, while the mean SPPB was 6.8 ± 3.2 (range 3–12). Regarding the nutritional and dysphagia assessment, the average MNA-SF was 11.1 ± 2.3 and the mean EAT-10 was 1.9 ± 3.4, but with a median value of 0. Significant differences were found between frail and non-frail patients in each parameter, except for the BMI (Table 2). Table 3 shows the differences in terms of positive responses to Sunfrail items in frail and robust individuals: questions 2, 3, and 5 showed significant differences between the two categories of individuals.

**Table 2:** Characteristics of study population

	Total (*n* = 235)	Frail (*n* = 210)	Robust (*n* = 25)	*p* value*
Age, median (IQR)	82 (77–87)	83 (77–88)	79 (73–81)	**0.002**
Gender (female), *n* (%)	109 (46.4)	105 (50.0)	4 (16.0)	**0.001**
BMI, median (IQR)	26.8 (24.1–29.8)	26.8 (23.7–29.7)	26.9 (24.6–30.0)	0.724
SPPB, median (IQR)	7 (4–9)	6 (4–9)	11 (11–12)	**< 0.001**
Sunfrail total score, mean ± SD	4.0 ± 1.6	4.1 ± 1.6	3.0 ± 1.3	**0.001**
Sunfrail physical domain score, mean ± SD	2.4 ± 1.2	2.5 ± 1.2	1.6 ± 1.0	**0.001**
Sunfrail cognitive domain positivity, *n* (%)	164 (69.8)	146 (69.5)	18 (72)	0.998
Sunfrail social score, median (IQR)	1 (0–1)	1 (0–1)	1 (0–1)	0.104
MMSE, median (IQR)	23.4 (19.2–26.3)	22.7 (18.7–26.0)	26.9 (25.2–28.4)	**< 0.001**
Grip Strength (impairment, EWGSOP 2 criteria), *n* (%)	128 (54.5)	122 (58.1)	6 (24.0)	**0.001**
MNA-SF, median (IQR)	12 (9–13)	11 (9–13)	14 (12–14)	**< 0.001**
EAT-10, median (IQR)	0 (0–2)	0 (0–2)	0 (0–0)	**0.006**
GDS-5, median (IQR)	1 (0–3)	1 (1–3)	0 (0–1)	**< 0.001**

*IQR* interquartile range, *BMI* body mass index, *SPPB* Short Physical Performance Battery, *MMSE* Mini Mental State Examination, *EWGSOP* European Working Group on Sarcopenia in Older People, *MNA-SF* Mini Nutritional Assessment Short Form, *EAT-10* Eating Assessment Tool 10 items, *GDS-5* Geriatric Depression Scale-5 items

*In bold—the significant *p* values

**Table 3:** Differences in positive responses to Sunfrail items in frail and robust individuals

	Total (*n* = 235) *n* (%)	Frail (*n* = 210) *n* (%)	Robust (*n* = 25) *n* (%)	*p* value* Fisher
1. Do you regularly take 5 or more medications per day?	140 (59.6)	127 (60.5)	13 (52)	0.518
2. Have you recently lost weight such that your clothes have become looser?	71 (69.8)	68 (32.4)	3 (12)	**0.039**
3. Your physical state made you walking less during the last year?	165 (70.2)	156 (74.3)	9 (36)	**< 0.001**
4. Were you visited by your family doctor during the past year?^a^	95 (40.4)	83 (39.5)	12 (48)	0.518
5. Have you fallen one or more times over the past year?	90 (38.3)	86 (40.9)	4 (16)	**0.016**
6. Have you experienced memory decline during the last year?	164 (69.8)	146 (69.6)	18 (62)	0.999
7. Do you feel lonely most of the time?	100 (42.9)	92 (44.2)	8 (32)	0.289
8. If necessary, can you count on someone close to you?^a^	5 (2.1)	3 (1.4)	2 (8)	0.089
9. Have you had any financial difficulties in facing dental care and health care costs during the last year?	102 (43.4)	96 (45.7)	6 (24)	0.053

*In bold the significant *p* values

^a^To the questions 4 and 8 were assigned one point for negative answers

### Diagnostic accuracy

**Fig. 1:**
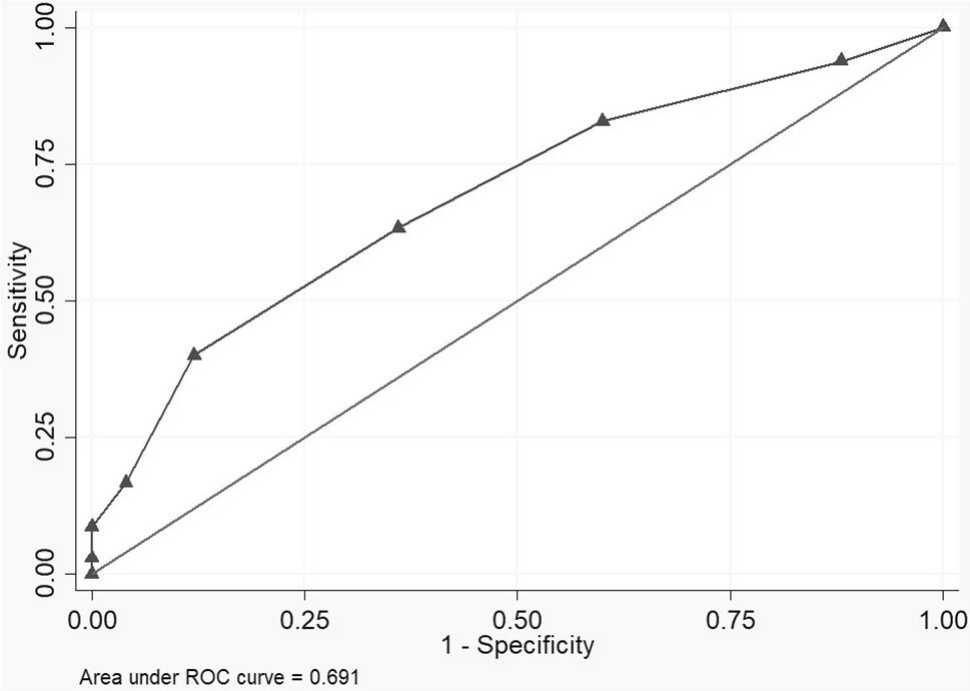
Diagnostic accuracy of the Sunfrail for the judgement of frailty by the CGA team

Using the CGA’s judgement of frailty as gold standard, the ROC analysis was conducted to verify the diagnostic accuracy and to identify possible cut-off scores. The ST total score was associated with the CGA’s judgement of frailty with an AUC of 0.691 (CI 95%: 0.591–0.791); according to the Youden index (YI), the best compromises between sensitivity and specificity were obtained with a cut-off score of 3 (sensitivity 83%, specificity 40%, YI: 0.23) and 4 (sensitivity 63%, specificity 64%, YI: 0.27) (Fig. 1). In particular, with a cut-off score of 3, the PPV was 21.7%, while the NPV was 92.1%; with a cut-off score of 4, the PPV was 94.7%, while the NPV was 17.2%.

### Construct validity

Table 4 shows the correlations between the ST and the CGA’s tests: all of these tests significantly correlated with the ST total score and the ST social domain. All tests correlated with the physical domain of the ST, with the exception of the MMSE. Finally, only MMSE and GDS-5 were significantly correlated with the cognitive domain of the ST.

**Table 4:** Correlations between SUNFRAIL tool total and its domains with SPPB, Handgrip, MMSE, GDS-5, MNA-SF, and EAT-10

	Sunfrail total		Sunfrail physical		Sunfrail cognitive		Sunfrail social	
	*r*	*p* value*	*r*	*p* value*	*r*	*p* value*	*r*	*p* value*
SPPB	− 0.335	**< 0.001**	− 0.339	**< 0.001**	− 0.023	0.728	− 0.196	**0.003**
Grip-Strength	− 0.323	**< 0.001**	− 0.277	**< 0.001**	− 0.026	0.698	− 0.272	**< 0.001**
MMSE	− 0.216	**0.001**	− 0.111	0.098	− 0.174	**0.009**	− 0.186	**0.005**
GDS-5	0.394	**< 0.001**	0.227	**< 0.001**	0.190	**0.004**	0.366	**< 0.001**
MNA-SF	− 0.425	**< 0.001**	− 0.367	**< 0.001**	− 0.097	0.147	− 0.296	**< 0.001**
EAT-10	0.306	**< 0.001**	0.330	**< 0.001**	− 0.001	0.988	0.162	**0.015**

*SPPB* Short Physical Performance Battery, *MMSE* Mini Mental State Examination, *GDS-5* Geriatric Depression Scale-5 items, *MNA-SF* Mini Nutritional Assessment Short Form, *EAT-10* Eating Assessment Tool-10 items

*In bold—the significant *p* values

### Discriminating power of the Sunfrail tool

We investigated the discriminating power of 5 questions of the ST against the CGA’s tests, and we found some significant differences. Individuals who answered “yes” to question n°2 presented lower values at the MNA-SF, indicating higher risk of malnutrition, and higher values at the EAT-10, consistent with more likely presence of dysphagia. The score of SPPB, the test that can better capture physical function, was lower in patients who answered “yes” to the question n°3, and in whom answering “yes” to the question n°5. As expected, we found lower MMSE values in persons who answered “yes” to the question n°6. Finally, participants who reported to feel lonely most of the time (question n°7) presented higher values on the GDS-5 (Table 5).

**Table 5:** Differences in the CGA based on responses to Sunfrail

	MNA-SF, mean ± SD	EAT-10, mean ± SD	SPPB, mean ± SD	MMSE, mean ± SD	GDS-5, mean ± SD
Have you recently lost weight such that your clothes has become looser?					
Yes	10.0 ± 2.4**	2.8 ± 4.4*			
No	11.6 ± 2.1**	1.5 ± 2.8*			
Your physical state made you walking less during the last year?					
Yes			6.1 ± 3.1**		
No			8.4 ± 2.8**		
Have you fallen one or more times over the past year?					
Yes			7.5 ± 3.2**		
No			5.7 ± 3.0**		
Have you experienced memory decline during the last year?					
Yes				22.1 ± 4.9*	
No				23.9 ± 3.9*	
Do you feel lonely most of the time?					
Yes					2.1 ± 1.4**
No					1.1 ± 1.1**

*p < 0.05, **p < 0.001

### Contribution to the prevention of falls

After an analysis with a stepwise logistic regression model, which used the positive answer to the question n°5 as dependent variable and the CGA’s physical tests (SPPB and Handgrip test) as independent variables, adjusted by the known possible confounders (sex, age, BMI, and MMSE), the only one which significantly correlated was the SPPB (OR: 0.839, CI 95%: 0.766–0.918, *p* value < 0.001) (data not shown). In this perspective, a low performance at the SPPB may represent a predictor of falls. On this assumption, the link between SPPB and age, gender, BMI, MMSE, and Grip-Strength was investigated, to verify if SPPB could be considered a proxy of a more in-depth physical evaluation, and each variable was significantly correlated with the SPPB (data not shown).

## Discussion

In the context of Frailty University-Hospital Lab, we found an AUC value close to 0.7, making the ST an easy-to-use tool [33] for the screening of frailty, but with a low diagnostic accuracy if compared to a CGA. This result is difficult to be compared with other instruments: many tools follow different, mono-domain, models of frailty (especially the clinical one), and in the most of the studies, the Criterion Validity has not been assessed, or has been through criteria which cannot be considered as a reasonable gold standard [14]. Both of the possible identified scores can be used as cut-off score for frailty, with similar predictive performances. However, as reported in previous studies [17], the main purpose of the ST is not to replace the CGA, but to identify the individuals who deserve CGA assessment. In this perspective, ST still represents a useful tool in the screening of frailty, thanks to its high discriminating power of false negative, as reported in literature [17]. Nevertheless, previous studies suggest to use a cut-off score of 3 [16].

Regarding the construct validity, all the multi-domain tests of the CGA significantly correlated with the total score of the ST, although with weak-moderate strength [34]. This suggests that this tool may be capable to capture the complexity of frailty, according to the holistic vision contained in the biopsychosocial model. Unexpectedly, the social domain of the ST showed significant correlations with all the CGA’s test, even the physical ones. This may be consistent with the evidence that social frailty can precede and/or lead to other domains of frailty [35]. Moreover, depression affects all domains of frailty including the physical function [36].

The ST showed a potential discriminating power, with significant mean differences in the CGA’s tests result. It is reasonable to assume that further studies should investigate if these 5 items (questions 2–3–5–6–7) can guide all the health care professional figures involved in the second-level assessment to prioritize frailty domains.

Regarding specific care path, the question n°5 was significantly associated with the SPPB, which was in turn correlated with other physical tests of the CGA related to falls. These results suggest a clinical pathway where the ST represents the first assessment level, to be carried out in the community, in the closely living context for the older persons. As previously mentioned, the ST can be administered by multiple professional figures, making it extremely adaptable to settings with unequally resources. If question n°5 turns out to be positive, the patient can be directed to an appropriately trained figure present in the community, i.e. a Community Health Nurse, and then assessed through the SPPB. If the administration of SPPB detects a deficit in the physical function, the patient can be directed towards a highly specialized CGA second level evaluation more likely available in the hospital setting. This process should improve the appropriateness of the geriatric evaluations, while increasing the operational efficiency of all the figures acting in the community. However, this approach brings to light the need for educational programs involving all the actors providing care to older individuals. Actually, the health response to frailty is mainly reactive, targeting its acute late events; for this reason, it is necessary to shift to a “proactive” model, instead to the “reactive” actual one [37], which obstacles the application of the discussed pathways. In fact, the primary care setting should include programs of screening and subsequent early interventions, to increase the therapeutic margin of these patients [38]. In this perspective, the Sunfrail may represent a first-line tool in a proactive, community-based, screening program [37].

### Limitations and strengths

This study presents some limitations.

Our sample represents a secondary care settings’ population, which is probably more selected than a primary care settings’ population, resulting in a selection bias, as can be seen from the higher prevalence of frail individuals. Moreover, our definition of frailty, although consistent with the bio-psycho-social definition of frailty [24], may have resulted in a very low threshold of frailty. This may have affected the estimation of the PPV and NPV.

Although the blindness of the two evaluations was accomplished, the ST was not directly administered, but the responses were extrapolated from the clinical information collected by routine-assessment.

However, by the direct comparison between ST and CGA and not another tool should ensure a greater reliability of the results, especially considering the bio-psycho-social nature of frailty. In fact, most instruments are mono-dimensional, and they are traditionally time-consuming.

## Conclusions

The Sunfrail tool is an easy-to-use instrument for the screening of frailty, consistent with the biopsychosocial model. Its questions may be used to guide a second level assessment, and to create operational care paths that include multiple settings. However, further studies with a prospective design are required to better investigate the performance of this tool.

## References

[1] Longobucco Y, Benedetti C, Tagliaferri S (2019). Proactive interception and care of frailty and multimorbidity in older persons: the experience of the European Innovation Partnership on Active and Healthy Ageing and the response of Parma Local Health Trust and Lab through European Projects. Acta Bio-medica Atenei Parmensis.

[2] Clegg A, Young J, Iliffe S (2013). Frailty in elderly people. Lancet.

[3] Sacha J, Sacha M, Soboń J (2017). Is it time to begin a public campaign concerning frailty and pre-frailty? A review article. Front Physiol.

[4] Collard RM, Boter H, Schoevers RA (2012). Prevalence of frailty in community-dwelling older persons: a systematic review. J Am Geriatr Soc.

[5] Gobbens RJ, van Assen MA, Luijkx KG (2011). Testing an integral conceptual model of frailty. J Adv Nurs.

[6] Sutorius FL, Hoogendijk EO, Prins BAH (2016). Comparison of 10 single and stepped methods to identify frail older persons in primary care: diagnostic and prognostic accuracy. BMC Fam Pract.

[7] Singer SJ, Burgers J, Friedberg M (2010). Defining and measuring integrated patient care: promoting the next frontier in health care delivery. Med Care Res Rev.

[8] van Kempen JAL, Schers HJ, Jacobs A (2013). Development of an instrument for the identification of frail older people as a target population for integrated care. Br J Gen Pract.

[9] Cesari M, Prince M, Thiyagarajan JA (2016). Frailty: an emerging public health priority. J Am Med Dir Assoc.

[10] Dedeyne L, Deschodt M, Verschueren S (2017). Effects of multi-domain interventions in (pre)frail elderly on frailty, functional, and cognitive status: a systematic review. Clin Interv Aging.

[11] Ng TP, Feng L, Nyunt MSZ (2015). Nutritional, physical, cognitive, and combination interventions and frailty reversal among older adults: a randomized controlled trial. Am J Med.

[12] Puts MTE, Toubasi S, Andrew MK (2017). Interventions to prevent or reduce the level of frailty in community-dwelling older adults: a scoping review of the literature and international policies. Age Ageing.

[13] Dent E, Kowal P, Hoogendijk EO (2016). Frailty measurement in research and clinical practice: a review. Eur J Intern Med.

[14] Sutton JL, Gould RL, Daley S (2016). Psychometric properties of multicomponent tools designed to assess frailty in older adults: a systematic review. BMC Geriatr.

[15] Adams WL, McIlvain HE, Lacy NL (2002). Primary care for elderly people: why do doctors find it so hard?. Gerontologist.

[16] Gobbens RJJ, Maggio M, Longobucco Y (2020). The validity of the Sunfrail tool: a cross-sectional study among Dutch community-dwelling older people. J Frailty Aging.

[17] Maggio M, Barbolini M, Longobucco Y (2020). A novel tool for the early identification of frailty in elderly people: the application in primary care settings. J Frailty Aging.

[18] Cardoso AF, Bobrowicz-Campos E, Couto F (2019). Feasibility, appropriateness and meaningfulness analysis of the Sunfrail tool to the European Portuguese population during cross-cultural adaptation process. Int J Evid Based Healthc.

[19] Tinetti ME, Speechley M, Ginter SF (1988). Risk factors for falls among elderly persons living in the community. N Engl J Med.

[20] Bennett C, Khangura S, Brehaut JC (2011). Reporting guidelines for survey research: an analysis of published guidance and reporting practices. PLoS Med.

[21] Burns KEA, Duffett M, Kho ME (2008). A guide for the design and conduct of self-administered surveys of clinicians. Can Med Assoc J.

[22] Kelley K, Clark B, Brown V (2003). Good practice in the conduct and reporting of survey research. Int J Qual Health Care.

[23] van Kempen JAL, Schers HJ, Melis RJF (2014). Construct validity and reliability of a two-step tool for the identification of frail older people in primary care. J Clin Epidemiol.

[24] Gobbens RJ, Luijkx KG, Wijnen-Sponselee MT (2010). Toward a conceptual definition of frail community dwelling older people. Nurs Outlook.

[25] Folstein MF, Folstein SE, McHugh PR (1975). "Mini-mental state". A practical method for grading the cognitive state of patients for the clinician. J Psychiatr Res.

[26] Frisoni GB, Rozzini R, Bianchetti A (1993). Principal lifetime occupation and MMSE score in elderly persons. J Gerontol.

[27] Guralnik JM, Ferrucci L, Pieper CF (2000). Lower extremity function and subsequent disability: consistency across studies, predictive models, and value of gait speed alone compared with the Short Physical Performance Battery. J Gerontol A Biol Sci Med Sci.

[28] Guralnik JM, Ferrucci L, Simonsick EM (1995). Lower-extremity function in persons over the age of 70 years as a predictor of subsequent disability. N Engl J Med.

[29] Ibrahim K, May C, Patel HP (2016). A feasibility study of implementing grip strength measurement into routine hospital practice (GRImP): study protocol. Pilot Feasibility Stud.

[30] Cruz-Jentoft AJ, Bahat G, Bauer J (2019). Sarcopenia: revised European consensus on definition and diagnosis. Age Ageing.

[31] Hoyl MT, Alessi CA, Harker JO (1999). Development and testing of a five-item version of the Geriatric Depression Scale. J Am Geriatr Soc.

[32] Tagliaferri S, Lauretani F, Pela G (2018). The risk of dysphagia is associated with malnutrition and poor functional outcomes in a large population of outpatient older individuals. Clin Nutr (Edinburgh, Scotland).

[33] Mandrekar JN (2010). Receiver operating characteristic curve in diagnostic test assessment. J Thorac Oncol.

[34] Akoglu H (2018). User's guide to correlation coefficients. Turk J Emerg Med.

[35] Makizako H, Shimada H, Doi T (2018). Social frailty leads to the development of physical frailty among physically non-frail adults: a four-year follow-up longitudinal cohort study. Int J Environ Res Public Health.

[36] Mulasso A, Roppolo M, Giannotta F (2016). Associations of frailty and psychosocial factors with autonomy in daily activities: a cross-sectional study in Italian community-dwelling older adults. Clin Interv Aging.

[37] Lauretani F, Longobucco Y, Ferrari Pellegrini F (2020). Comprehensive model for physical and cognitive frailty: current organization and unmet needs. Front Psychol.

[38] Di Bari M, Profili F, Bandinelli S (2014). Screening for frailty in older adults using a postal questionnaire: rationale, methods, and instruments validation of the INTER-FRAIL study. J Am Geriatr Soc.

